# Differential effects of alcohol‐drinking patterns on the structure and function of the brain and cognitive performance in young adult drinkers: A pilot study

**DOI:** 10.1002/brb3.2427

**Published:** 2021-11-22

**Authors:** Xiaobing Guo, Tongjun Yan, Min Chen, Xiaoyan Ma, Ranli Li, Bo Li, Anqu Yang, Yuhui Chen, Tao Fang, Haiping Yu, Hongjun Tian, Guangdong Chen, Chuanjun Zhuo

**Affiliations:** ^1^ The National Clinical Research Center for Mental Disorders and Beijing Key Laboratory of Mental Disorders Beijing Anding Hospital Capital Medical University Beijing China; ^2^ Advanced Innovation Center for Human Brain Protection Capital Medical University Beijing China; ^3^ Department of Psychiatry 904th Hospital of PLA Changzhou Jiangsu China; ^4^ Institute of Mental Health Jining Medical University Jining China; ^5^ Department of Alcohol Dependence Management Tianjin Anding Hospital Tianjin Medical University Clinical Hospital of Mental Health Tianjin China; ^6^ Tianjin Anding Hospital Tianjin Mental Health Center Key Laboratory of Psychiatry Neuroimaging‐Genetics and Co‐morbidity (PNGC_Lab) of Tianjin Medical University Clinical Hospital of Mental Health Nankai University Affiliated Tianjin Anding Hospital Tianjin China; ^7^ Department of Psychiatry Tianjin Kangtai Mental Health Hospital Tianjin China; ^8^ Key Laboratory of Real Time Brain Circuits Tracing of Neurology and Psychiatry (RTBNB_Lab) Tianjin Fourth Center Hospital Tianjin Medical Affiliated Tianjin Fourth Central Hospital Nankai University Affiliated Tianjin Fourth Center Hospital Tianjin China; ^9^ Department of Alcohol Dependence Management Wenzhou Seventh Peoples Hospital Wenzhou China

**Keywords:** alcohol, alcohol‐drinking pattern, brain features, cognitive impairment, magnetic resonance imaging

## Abstract

**Introduction:**

This study was aimed to determine how different patterns of alcohol consumption drive changes to brain structure and function and their correlation with cognitive impairments in young adult alcohol drinkers.

**Methods:**

In this study, we enrolled five groups participants and defined as: long‐term abstinence from alcohol (LA), binge drinking (BD), long‐term low dosage alcohol consumption but exceeding the safety drinking dosage (LD), long‐term alcohol consumption of damaging dosage (LDD), and long‐term heavy drinking (HD). All participants underwent magnetic resonance imaging (MRI) and functional MRI (fMRI) to acquire data on brain structure and function, including gray matter volume (GMV), fractional amplitude of low‐frequency fluctuation (fALFF), regional homogeneity (ReHo), functional connectivity (FC), and brain network properties. The cognitive ability was evaluated with the California Verbal Learning Test (CVLT), intelligence quotient (IQ), and short delay free recall (SDFR).

**Results:**

Compared to LA, GMV significantly decreased in the brain regions in VN, SMN, and VAN in the alcohol‐drinking groups (BD, LD, LDD, and HD). ReHo was significantly enhanced in the brain regions in VN, SMN, and VAN, while fALFF significantly increased in the brain regions in VN and SMN. The number of intra‐ and inter‐modular connections within networks (VN, SMN, sensory control network [SCN], and VAN) and their connections to other modules were abnormally changed. These changes adversely affected cognition (e.g., IQ, CVLT, SDFR).

**Conclusion:**

Despite the small sample size, this study provides new evidence supporting the need for young people to abstain from alcohol to protect their brains. These findings present strong reasoning for updating anti‐alcohol slogans and guidelines for young people in the future.

## Graphical abstract

The alcohol‐drinking patterns exert differential effects on the brain structure and function, and cognitive performance in young adult drinkers.

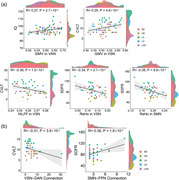

## 1 INTRODUCTION

Chronic alcohol use and misuse causes structural and functional impairments to the brain, leading to alcohol‐related brain damage (ARBD; Aziz, 2014; Horton et al., 2015). The effects of drinking alcohol on the structure and function of the brain are more serious in young adults compared to middle‐aged and older adults (Giedd et al., 1999; Koob, 2018; Shokri‐Kojori et al., 2017). Consequently, many studies have extensively investigated the effects of drinking alcohol on the structure and function of the brain in the young adult population. For instance, Carrino et al. (2021) reported that multiple brain cortical regions (including the right orbitofrontal, right temporal pole, and left lateral occipital) were affected by young adults drinking alcohol, and were mainly expressed as changes to cortical gyrification. Morris et al. (2019) showed that young adult drinkers had a thinner whole‐brain cortical thickness compared to healthy controls. Drinking alcohol by young adults also causes microstructural impairments to white matter (WM) in the whole brain, especially in the fornix and corpus callosum (Boness et al., 2020).

In parallel to impacting brain structure, alcohol negatively impacts brain function in young adults who drink alcohol. For example, Veer et al. (2019) showed that the functional connectivity (FC) of the nucleus accumbens at rest is associated with alcohol consumption in young adult males. Ware et al. (2021) demonstrated that the FC of the attention networks is altered in young adult drinkers. Jia et al. (2021) found that drinking alcohol affects the neural network involved in regulating the medial orbitofrontal cortex and dorsal periaqueductal gray matter (GM) in young adults. Collectively, alcohol drinking causes substantial structural and functional impairments to the brain of young adult drinkers, with changes generally occurring in the frontal, temporal, parietal and occipital lobes.

Alcohol use and misuse can cause both physical brain damage and psychosocial impairment (De Santis et al., 2019; Osna et al., 2020; Robert et al., 2020; Sullivan et al., 2018). Unfortunately, global alcohol consumption continues to rise, particularly with respect to problem drinking in young people (Pantani et al., 2020; Windle, 2020). To reduce alcohol use and associated consequences in young adults, the World Health Organization (WHO) created the slogan: “less alcohol, the better” (https://www.who.int/topics/alcohol_drinking/zh/). Due to growing issues with alcohol consumption, many countries have implemented national strategies and measures to reduce alcohol use and misuse, particularly in young adult alcohol drinkers (Bowring et al., 2012; Rehm & Patra, 2012). For example, Canada's low‐risk alcohol‐drinking guidelines (Stockwell et al., 2012) state that late teens (aged 16–19) and young adults (aged 18−24) should not exceed two to three drinks per day (females and males, respectively). These guidelines warn young people that drinking above the stated limit increases the risk of physical and psychosocial impairments to the brain (Stockwell et al., 2012). These guidelines might be misleading in warning that serious consequences are only incurred over the alcohol consumption limit, with no impact below the limit. Hence, some people might adopt “safe” drinking patterns, even though the long‐term intake of two to three drinks could cause alcohol‐related anhedonia. Therefore, these guidelines have been subject to debate since coming into effect (Thompson et al., 2012).

It has been demonstrated that chronic alcohol use and misuse causes structural and functional impairments to the brain, leading to ARBD (Aziz, 2014; Horton et al., 2015). It is particularly urgent to reduce the risk of ARBD in young adults (Hermens et al., 2013). ARBD in young adults usually causes more serious impairments than in middle‐aged and older adults (Giedd et al., 1999; Koob, 2018; Shokri‐Kojori et al., 2017). ARBD usually involves structural or functional impairments in regions of the brain that have key roles in cognitive processing. Abnormal changes in these regions are usually associated with cognitive disruptions (Le Berre et al., 2017; Luo et al., 2020; Oberlin et al., 2018; Pandey et al., 2018; Wesley et al., 2017; Zahr et al., 2017; Zehra et al., 2019). However, research on ARBD and the associated cognitive impairments in alcohol drinkers with no symptoms of alcohol use disorder remains limited, especially in young adults. Consequently, it is unclear whether the degree of ARBD and cognitive damage are associated with different patterns of alcohol consumption and, more importantly, whether “safe” alcohol‐drinking patterns cause ARBD and cognitive damage. Knowledge of how ARBD and cognitive damage differs between “safe” versus other types of alcohol‐drinking patterns remains unclarified.

Alcohol‐drinking patterns have been classified into five categories by the National Institute of Alcohol and Alcoholism (NIAAA) and WHO (Kerr, 2010; Naimi et al., 2003; Patrick et al., 2017). These categories include: (1) long‐term abstinence from alcohol (LA); (2) binge drinking (BD), intake of an excessive, large amount of alcohol over a short period of time (within a 2‐h time period on one occasion) with no intention to stop drinking (two to three times per month with ≥4 and 5 drinks for females and males, respectively); (3) long‐term damaging alcohol drinking that exceeds the safe drinking dosage recommend by WHO (LD), with alcohol intake of <4 to ≥3 drinks and <5 to ≥4 drinks per day for females and males, respectively; (4) long‐term low dosage drinking, involving the consumption maximum two drinks of alcohol per day for both females and males (LDD); and (5) long‐term heavy drinking (HD), alcohol intake ≥5 drinks per day, regardless of sex. Previous studies reported that alcohol‐drinking patterns are associated with different risks to ARBD, physiological alcohol‐related diseases, financial burdens of ARBD (Esser et al., 2020; Hendriks, 2020; Liu, 2020; Silveira et al., 2020). Drinking alcohol impairs specific brain regions, including the occipital lobe, parietal lobe, frontal cortex, temporal lobe, cingulate cortex, and thalamus. These regions are the major components of the sensorimotor cortex network (SMN), dorsal attention network (DAN), ventral attention network (VAN), and visual network (VN), which play a pivotal role in cognitive processing (Chumin et al., 2019; Kamarajan et al., 2020; Silveira et al., 2020; Song et al., 2020). Several identified deficits to working memory and executive function, impairments of executive function, visuo‐perceptual difficulties, profound memory impairment, and impaired spatial memory in young adult alcohol drinkers, which had corresponding damaged brain regions (e.g., frontal cortical shrinkage, hippocampus shrinkage and cerebellar cell loss; Lindgren et al., 2019; Mira et al., 2019; Nunes et al., 2019; Sullivan & Pfefferbaum, 2019). Thus, memory function is likely the main specific domain of cognitive functioning that is affected the most (Lindgren et al., 2019; Mira et al., 2019; Nunes et al., 2019; Sullivan & Pfefferbaum, 2019). However, to the best of our knowledge, few studies have assessed the specific relationship between different cognitive impairments and different drinking patterns in young adults who drink alcohol.

Existing studies support that there is an association between alcohol‐drinking patterns and different structural and functional impairments to the brain, especially in young adults who drink alcohol (Kim et al., 2020; Liu et al., 2018; Mashhoon et al., 2014; Tu et al., 2018; Zhao et al., 2021). Mashhoon et al. (2014) reported that past and recent BD are associated with thinner frontal cortical thickness of the right mid‐anterior cingulate cortex and left posterior cingulate cortex in young adults. Zhao et al. (2021) suggested that heavy alcohol drinking is associated with impairments to the microstructural integrity of brain WM in adolescent heavy drinkers. Simultaneously, Liu et al. (2018) reported the aberrant amplitude of low‐frequency fluctuations (ALFF) in the prefrontal–parietal–cerebellar circuit in patients with alcohol dependence. Tu et al. (2018) found that alcohol‐dependent patients exhibited higher regional homogeneity (ReHo) in the right superior frontal gyrus (SFG), bilateral medial frontal gyrus, left precentral gyrus (PG), bilateral middle temporal gyrus, and right inferior temporal gyrus (ITG), as well as in the lower ReHo in the right cerebellum posterior lobe, left rectal gyrus, and right cluster of pons and cerebellum anterior lobe. Kim et al. (2020) reported that FC is altered in the left prefrontal–parietal–occipital midline circuits of BD college students, with these changes being associated with their visual memory. Correas et al. (2015) found that both structural and functional alterations occur in the frontal and parietal lobes of young binge drinkers, and that the dysfunction of frontal–parietal network subsequently affects the performance of executive function. These studies support the hypothesis that different drinking patterns cause different structural and functional impairments to the brain.

Here, we conducted a pilot study using multiple magnetic resonance imaging (MRI) techniques to establish how different alcohol‐drinking patterns in young adults alter the structure, function, and connectome of the brain, and contribute to cognitive impairments. We hypothesized that different alcohol‐drinking patterns are associated with differential impairments to the structure and function of the brain, thereby leading to different degrees of cognitive impairments. The findings obtained through conducting this study may have broader implications for future updating anti‐alcohol slogans and guidelines.

## 2 MATERIALS AND METHODS

### 2.1 Participants and study design

This pilot study spanned 4 years between June 2016 and July 2020. Participants were prospectively recruited from the Traffic Control Bureau (Tianjin, China) and a community near the Tianjin Kangtai Hospital (Tianjin, China). During enrollment, the following inclusion criteria were used: (1) physically and mentally healthy individuals with no evidence of disease; these individuals were considered to be physically and mentally healthy based on initial physical and mental screening tests conducted by physicians and psychiatrists; (2) history of alcohol intake assessed based on self‐reporting, and confirmed by their guardian and medical records; (3) complete information on the quantity and pattern of alcohol intake within the last 6 months; (4) intelligence quotient (IQ) ≥80 as assessed by psychological testing; and (5) routine blood and biochemical indices in the normal range based on laboratory examination of blood and urine samples. Individuals who had the following conditions were excluded from this study: (1) history of alcohol dependence based on the self‐report, and confirmed by their guardian; (2) history of other substance use based on a urine test; (3) history of cigarette‐smoking based on the self‐report and confirmation of their guardian; (4) history of mental disorder, neurological disease, or traumatic brain injury that caused the loss of consciousness for more than 3 min based on the self‐report and medical records; (5) current or history of tumor, endocrine nutrition metabolism disease, or circulatory system diseases based on the self‐report and medical records; and (6) presence of anxiety, sleep disorder, or depressive symptoms based on DSM‐IV criteria and scales following examination by two experienced psychiatrists. Participants with long‐term (≥4 years) abstinence from alcohol were prospectively enrolled from the Traffic Control Bureau (Tianjin, China), who had been detained and legally prohibited from drinking alcohol due to traffic violations in major traffic accidents. Until enrollment, participants had no opportunity to drink alcohol, because they were restrictedly administrated in the Traffic Control Bureau. Therefore, the participants maintained complete abstinence from alcohol, with these samples being considered as “pure” samples of the LA. At the time of enrollment, participants were generally healthy based on a physical and psychological examination at the Traffic Control Bureau.

Forty‐five participants were enrolled in this study, of which all were right‐handed, as defined by the Edinburgh Handedness Inventory (EHI; Christman et al., 2015). The enrolled participants were placed in five categories based on alcohol‐drinking patterns (e.g., intensity and frequency) as defied by the NIAAA: (1) LA, long‐term (≥4 years) abstinence from alcohol (*n* = 10); (2) BD, an excessive, large amount of alcohol in a short‐period of time (within a 2‐h period on one occasion) with no intention to stop drinking, with this pattern being maintained for ≥4 years (*n* = 9); (3) LD, long‐term (≥4 years) drinking of alcohol over the safe dosage recommend by WHO, but not exceeding the dosage for heavy drinking (< 3 drinks per day for females and <4 drinks per day for males; *n* = 8); (4) LDD, long‐time (≥4 years) drinking low doses of alcohol (<2 drinks per day regardless of sex; *n* = 7); and (5) HD, long‐term (≥4 years) drinking high doses of alcohol (≥5 drinks per day, regardless of sex; *n* = 11). Social–demographical and psychosocial characteristics and MRI data (e.g., structural and functional brain imaging and cognition measurements) were acquired for each participant and compared across groups.

All participants provided signed informed content. This study was reviewed and approved by the Ethics Committee of the participating hospitals.

### 2.2 Multiple MRI data acquisition

MRI examinations were performed with a 3.0 T scanner (Vision, Siemens Medical system, Germany). MRI data of the brain structure were acquired using specific parameters and protocols. The scanning protocol included a 3D T1‐weighted sequence that had been previously established as a standard routine in our study center (Tianjin Fourth Center Hospital). The parameters for the MRI examinations and data acquisition included: (1) Sagittal 3D T1‐weighted images: repetition time (TR), 8.2 ms; echo time (TE), 3.2 ms; inversion time (TI), 450 ms; flip angle (FA), 12°; field of view (FOV), 256 mm × 256 mm; matrix, 256 × 256; slice thickness, 1 mm, no gap; 188 sagittal slices; acquisition time, 250 s; (2) Resting‐state BOLD images using a gradient‐echo single‐shot echo‐planar imaging (GRE‐SS‐EPI) sequence: TR/TE, 2000/45 ms; FOV, 220 mm × 220 mm; matrix, 64 × 64; FA, 90°; slice thickness, 4 mm; gap, 0.5 mm; 32 interleaved transverse slices; 180 volumes; acquisition time, 370 s. All participants had to keep their eyes closed, stay awake, and move as little as possible during MRI imaging.

### 2.3 Structural MRI data processing

All structural MRI data were acquired with a controlled head motion of below 2 cm or rotated 2°, and were adjusted for age, gender, and level of education. MRI data on gray matter volume (GMV) was processed based on CAT12 (CAT12 (http://dbm.neuro.unijena.de/cat) as previously reported. In brief, the MRI data were normalized to MNI space, resliced to 3 mm^3^, and segmented into GM, WM, and cerebral spinal fluid (CSF). The GM images were smoothed with a FWHM of 6 × 6 × 6 mm^3^ Gaussian filter. Based on Brainnetome 246 regions (including subcortex nucleus; Ashburner, 2007; Ashburner & Friston, 2005; Rajapakse et al., 1997; Tohka et al., 2004), the mean GMV was calculated for the brain regions. The mean GMV in the eight networks of the brain as published by Thomas Yeo (Thomas Yeo's eight networks; Kashyap et al., 2019; Wang et al., 2019), including the subcortex nucleus were calculated for each network in the present study.

The MRI data on the fractional amplitude of low‐frequency fluctuations (fALFFs) were processed on SPM12 (http://www.fil.ion.ucl.ac.uk/spm). In brief, the first 10 volumes, slice timing, realignment, and normalization were delineated into the standard MNI space using data resliced to 3 mm^3^. Twenty‐four motion parameters were regressed out based on a linear drift, CSF, and WM signals. fALFF was estimated ([0.01–0.08 Hz]/[0–0.25 Hz]). FWHM was smoothed as 6 × 6 × 6 mm^3^ (Zou et al., 2008). Based on the Brainnetome of 246 regions, including the subcortex nucleus, the mean fALFF was calculated for each brain region. The mean fALFF was calculated for each network of the Brainnetome, including the subcortex nucleus (Wang et al., 2019).

MRI data on potential ReHo were processed using SPM12 (https://brant.brainnetome.org/en/latest/SPON_ReHo.html). In short, the first 10 volumes were deleted, slice‐time corrected, realigned, and normalized to the standard MNI space. The data were then resliced to 3 mm^3^. Twenty‐four motion parameters were regressed out, along with linear drift, CSF, and WM signals. The parameters were then filtered (0.01−0.08 Hz). ReHo was estimated with REST software and smoothed with an FWHM of 6 × 6 × 6 mm^3^ (Ma et al., 2020). The mean ReHo was calculated for each brain region based on the 246 regions of the Brainnetome, including the subcortex nucleus. The mean ReHo was specifically calculated for each network based on Thomas Yeo's eight networks, including the subcortex nucleus (Wang et al., 2019).

### 2.4 Processing functional MRI data for FC

When processing the regression of FC, we also regressed out mean frame‐wise displacement (FD; Küblböck et al., 2014). For FC, this was based on SPM12. The first 10 volumes were removed, slice timed, realigned, normalized to the standard MNI space, and resliced to 3 mm^3^. Twenty‐four motion parameters were regressed out, along with linear drift, CSF and WM signals. The parameters were then filtered (0.01−0.08 Hz) and smoothed with a FWHM of 6 × 6 × 6 mm^3^. Based on the 246 regions of Brainnetome, (including the subcortex nucleus), the FC matrix (246 matrix) and binary FC matrix were calculated. The most 1% positive FC was retained (Sui et al., 2018). The network properties were based on the software package of the Brain Connectivity Toolbox (Meunier et al., 2020). We first calculated the modularity of FC, Q (Tohka et al., 2004) based on the binary FC matrix, with no differences being detected among groups. The binary FC matrix (Owen et al., 2016) was then corresponded to the eight networks of Thomas Yeo through calculating the module segregation index (MSI; Hsu et al., 2019). Finally, we calculated the number of intranetwork connections and internetwork connections of the eight Thomas Yeo networks (Liégeois et al., 2019).

### 2.5 Assessment of cognitive ability

All participants completed cognitive ability tests. The California Verbal Learning Test (CVLT) was used to obtain more detailed memory assessments in participants with alcohol abuse (Heirene et al., 2018; Le Berre et al., 2017; Mahmood et al., 2010; Rehm et al., 2003). Subjects with different alcohol‐drinking patterns were validated through comprehensively assessing their cognitive ability (memory and verbal learning).

### 2.6 Statistical analysis

Statistical analysis was conducted using SPSS 21.0 statistical software (SPSS Chicago, IL, USA). To comparatively analyze the two groups, we conducted a permutation test 5000 times. Analysis of variance (ANOVA) was used to compare the difference between the five groups. Before the ANOVA analysis, we conducted a permutation test 5000 times to comparatively analyze the two groups, during which we controlled the FD by setting it as an independent variable. Correlations of CVLT/IQ with GMV, fALFF, ReHo, FC, and network properties were conducted. All *p* values were Bonferroni corrected, two‐sided. Note that *p* < .05 was considered to be statistically significant (Martínez‐Heras et al., 2020).

## 3 RESULTS

### 3.1 Social–demographical and psychosocial characteristics of the study subjects

Forty‐five participants were enrolled in this study, and were classified into five groups based on their alcohol‐drinking patterns (e.g., frequency, intensity): LA group (*n* = 10; *n* = 7 males and *n* = 3 females), BD group (*n* = 9; *n* = 6 males and *n* = 3 females), LD group (*n* = 8; *n* = 5 males and *n* = 3 females), LDD group (*n* = 7; *n* = 7 males), and HD group (*n* = 11; *n* = 7 males, and *n* = 4 females). The social–demographical characteristics (e.g., age, gender, educational levels), and psychosocial features are summarized in Table 1. There were no significantly differences in age, gender, educational level, or psychosocial characteristics between the groups (*p *> .05).

**TABLE 1:** Baseline social–demographical and psychosocial characteristics of the participants

Characteristics	LA(*n* = 10)	BD(*n* = 9)	LD(*n* = 8)	LDD(*n* = 7)	HD(*n* = 11)	*p*
Age	24.90 ± 1.60	23.89 ± 2.89	24.25 ± 1.91	24.43 ± 3.21	24.27 ± 2.24	.92
Gender (M/F)	7/3	6/3	5/3	7/0	7/4	.50
Education	14.60 ± 1.65	14.89 ± 1.76	14.75 ± 1.83	14.71 ± 1.89	14.18 ± 1.89	.92
Drink alcohol time‐4	0 ± 0	3.56 ± 2.46	4.38 ± 2.13	4.86 ± 1.77	4.73 ± 3.64	2.77 × 10^−4^
Total drinks in the last 6 months	0 ± 0	4533 ± 1526	695 ± 232	260 ± 78	1536 ± 456	4.84 × 10^−16^
History of family with alcohol heavy drinks (yes/no)	8/2	5/4	6/2	3/4	8/3	.50
IQ‐7	95.70 ± 8.00	95.67 ± 5.29	95.88 ± 5.96	94.43 ± 4.76	92.18 ± 2.60	.53
CVLT (learning trials correct response)	13.80 ± 0.92	9.22 ± 2.68	10.50 ± 1.69	7.86 ± 1.07	8.64 ± 1.69	3.42 × 10^−8^
SDFR	106.20 ± 15.71	86.56 ± 13.21	81.25 ± 11.29	85.71 ± 16.77	67.45 ± 15.77	2.21 × 10^−5^
Mean FD	0.060 ± 0.023	0.066 ± 0.019	0.093 ± 0.044	0.056 ± 0.018	0.065 ± 0.020	.0508

Abbreviations: BD, binge drinking; CVLT, the California Verbal Learning Test; F, female; FD, functional connectivity; HD, long‐term heavy drinking; IQ, intelligence quotient; LA, long‐term abstinence of alcohol; LD, long‐term alcohol drinking over safety drinking dosage; LDD, long‐term low dosage alcohol drinking; M, male; SDFR, short‐delay free recall.

### 3.2 Differences of GMV in the study subjects with different alcohol‐drinking patterns

**FIGURE 1:**
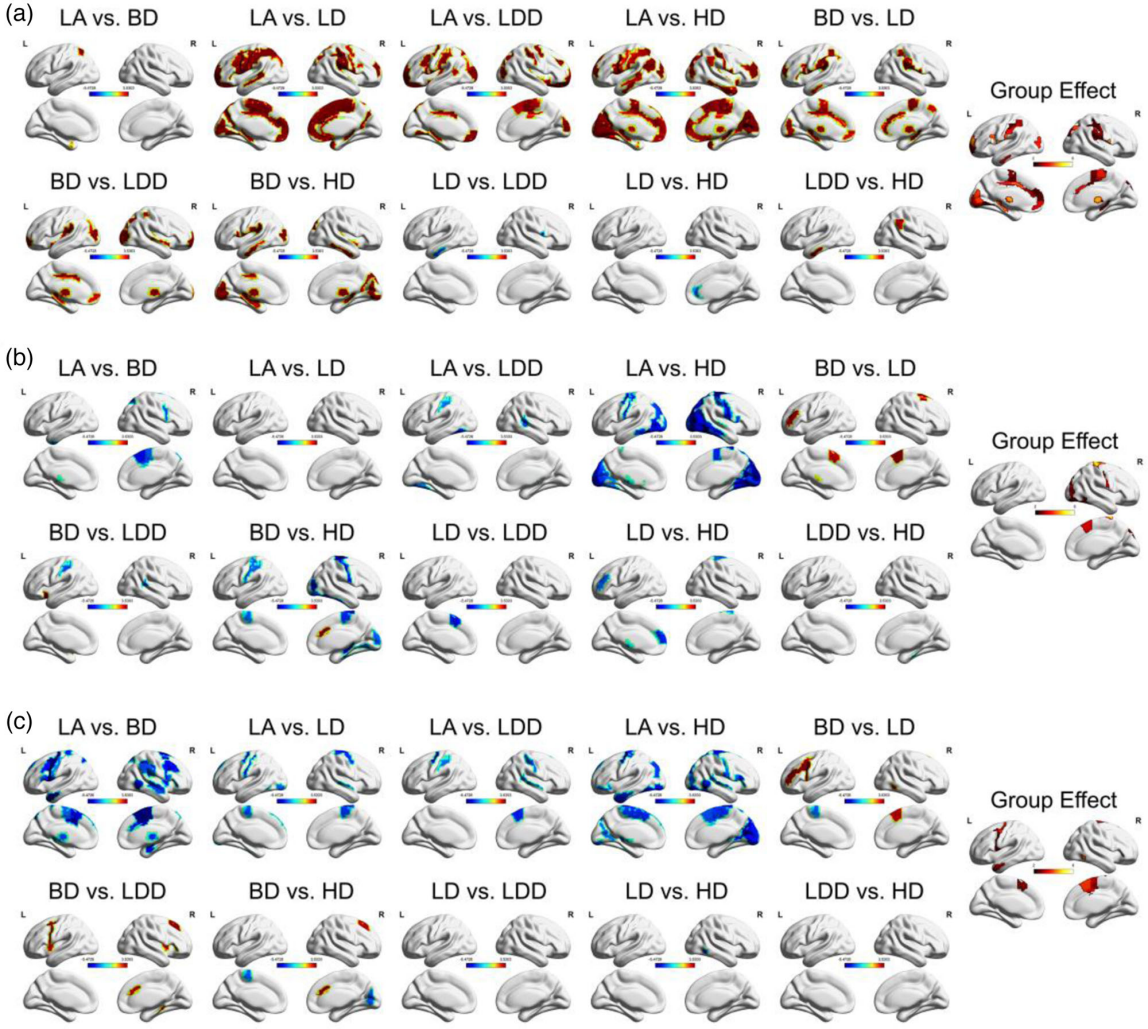
Structural and functional differences in the brain among the five alcohol‐drinking groups. (a) Differences in gray matter volume (GMV) among the five groups. Warm colors denote the regions where GMV was higher in the left panel versus the right panel, while cool colors indicate the regions where GMV was lower in the left panel versus the right panel. For instance, GMV was significantly greater in LA compared to HD, with abnormally low GMV in HD. (b) Functional amplitude with low‐frequency fluctuations (fALFF) among the five drinking groups. Differences fALFF among the five groups (LA, BD, LD, LDD, and HD) were determined. Warm colors indicate regions where fALFF was higher in the left panel versus the right panel, while cool colors indicate the regions where fALFF was lower in the left panel versus the right panel. fALFF was significantly lower in LA compared to HD group, with the hyper‐functioning of the brain likely compensating for lower GMV in HD. (c) Differences in regional homogeneity (ReHo) among the five groups (LA, BD, LD, LDD, and HD). Warm colors indicate regions where ReHo was greater in the left panel versus the right panel, while cool colors indicate regions where ReHo was lower in the left panel versus the right panel. For instance, ReHo was significantly lower in LA compared to HD, with the hyperfunctioning of the brain likely compensating for lower GMV in HD. Pair‐wise comparisons were made among the five groups (LA, BD, LD, LDD, and HD) in (a), (b), and (c) using a permutation test repeated 5000 times (*p* < .05). Group effect was determined by analysis of variance (ANOVA; *p* < .05)

The GMV of the groups with different drinking patterns is shown in Figure 1a. GMV altered significantly among the five groups (Group effect, with 5000 permutations, *p *< .05). Abnormal changes were detected in the five alcohol‐drinking groups (LA, BD, LD, LDD, HD), mainly in the medial occipital lobe, postcentral gyrus, sensorimotor cortex, premotor areas, intraparietal sulcus, paracentral lobule, anterior cingulate gyrus, middle cingulate gyrus, thalamus, and hippocampus (Figure 1a; group effect, with 5000 permutations, *p *< .05). GMV abnormalities had different profiles in pairwise comparisons (combinations of the five groups, with 5000 permutations). GMV in the inferior parietal lobe and hippocampus head was significantly lower in the BD group compared to the LA group (Figure 1a, *p *< .05). GMV in the medial occipital lobe, anterior cingulate gyrus, middle cingulate gyrus, paracentral lobule, sensorimotor cortex, and intraparietal sulcus was significantly lower in the LD group compared to the LA group (Figure 1a, *p *< .05). GMV in the paracentral lobule, premotor area, postcentral gyrus, frontal pole, and occipital pole was significantly lower in the LDD group compared to the LA group (Figure 1a, *p *< .05). GMV in the medial occipital lobe, paracentral lobule, sensorimotor cortex, intraparietal sulcus, and thalamus was significantly lower in the HD group compared to the LA group (Figure 1a, *p *< .05). GMV in ITG and supramarginal gyrus was significantly lower in the HD group compared to the LD group (Figure 1a, *p *< .05). GMV was significantly lower in the anterior cingulate cortex in the LD group compared to LDD (Figure 1a, *p *< .05).

### 3.3 Differences in the functional amplitude of low‐frequency fluctuations in participants with different alcohol‐drinking patterns

fALFF primarily differed in the supplementary motor area (SMA) and precentral gyrus among the five groups (Figure 1b; Group effect, with 5000 permutations, *p *< .05). fALFF in the superior parietal lobule, inferior frontal gyrus, and SMA was significantly greater in the BD group compared to the LA group (Figure 1b, *p *< .05). There was no significant difference between LA and LD (Figure 1b, *p *> .05). fALFF in the posterior central gyrus and superior temporal sulcus was significantly greater in the LDD group compared to the LA group (Figure 1b, *p *< .05). fALFF in the PG, postcentral gyrus, occipital lobe, and ITG was significantly greater in the HD group compared to the LA group (Figure 1b, *p *< .05). fALFF in the middle frontal gyrus, SMA, and thalamus was significantly greater in the BD group compared to the LD group (Figure 1b, *p *< .05). Reduction of fALFF in the postcentral gyrus and superior temporal sulcus was significantly lower in the BD group compared to the LDD group (Figure 1b, *p *< .05). fALFF in the central sulcus, postcentral gyrus, paracentral lobule, hippocampus, cuneus, and fusiform gyrus was significantly lower in the BD group compared to the HD group (Figure 1b, *p *< .05). fALFF in the SMA was significantly lower in the LD group compared to the LDD group (Figure 1b, *p *< .05). fALFF in the middle frontal gyrus, PG and thalamus was significantly lower in the HD group compared to the LD group (Figure 1b, *p *< .05). There was no significant difference between the LDD and HD groups (Figure 1b, *p *> .05).

### 3.4 Differences in the ReHo of participants with different alcohol‐drinking patterns

ReHo mainly differed in the PG, SMA, and ITG among the five groups (Figure 1c, group effect, with 5000 permutations, *p *< .05). Pairwise comparisons among the five groups revealed that ReHo was significantly greater in SMA, premotor cortex, PG, middle frontal gyrus, inferior frontal gyrus, hippocampus, and thalamus in BD compared to LA (Figure 1c, LA vs. BD, *p *< .05). ReHo was significantly higher in the PG, paracentral lobule, and inferior occipital gyrus of LD compared to LA (Figure 1c, LA vs. LD, *p *< .05). ReHo was higher in the PG, postcentral gyrus, SMA, and superior temporal sulcus of LDD compared to LA (Figure 1c, LA vs. LD, *p *< .05). ReHo was significantly higher in the PG, paracentral lobule, SMA, medial occipital lobe, middle frontal gyrus, superior temporal gyrus, and ITG in HD compared to LA (Figure 1c, LA vs. HD, *p *< .05). ReHo was higher in the middle frontal gyrus, and paracentral lobule in BD compared to LD, but was lower in the SMA (Figure 1c, LA vs. HD, *p *< .05). ReHo was higher in the PG, SFG, anterior insula, middle cingulate gyrus, and hippocampus in LDD compared to BD (Figure 1c, BD vs. LDD, *p *< .05. ReHo was higher in the paracentral lobule, and medial occipital lobe in HD compared to BD, but was lower in middle cingulate gyrus and middle frontal gyrus. There was no significant difference between LD and LDD (Figure 1C, LD vs. LDD) or HD and LDD (Figure 1c, LDD vs. HD). ReHo was higher in the ITG of HD compared to LD (Figure 1c, LD vs. HD, *p *< .05).

### 3.5 Brain network characteristics of participants with different alcohol‐drinking patterns

**FIGURE 2:**
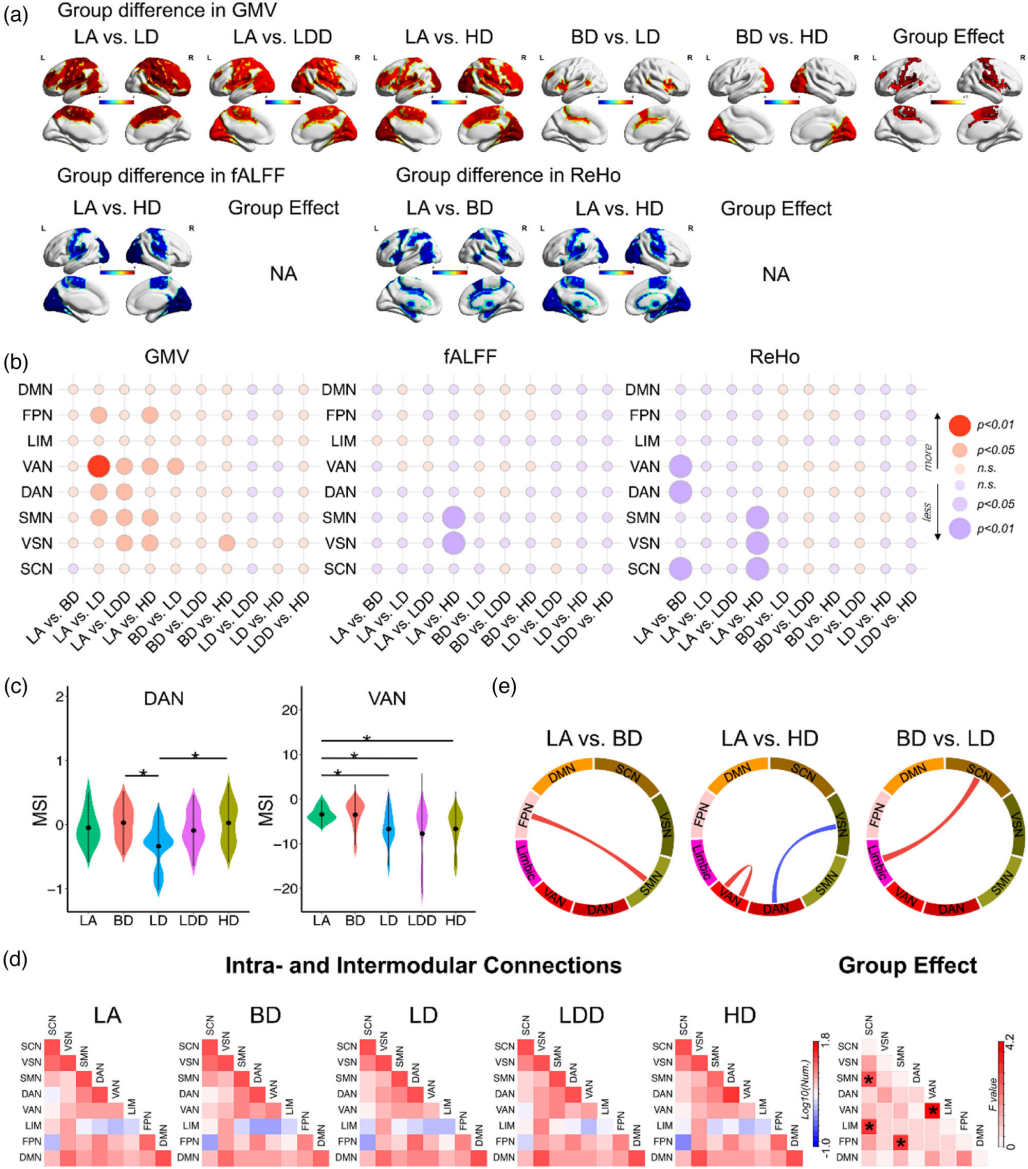
Profiles of the brain networks based on the eight networks of Thomas Yeo among the five alcohol‐drinking groups. (a) Differences in the structure and function of the brain, including GMV, fALFF, and ReHo within the brain networks of the five drinking groups. (b) Matrix scatterplot showing the differences in the brain networks of the groups based on the eight networks of Thomas Yeo (DMN, FPN, LIM, VAN, DAN, SMN, visual network [VSN], and SCN) for GMV, fALFF, and ReHo. Matrix plot presented the scatterplots of each X (difference in drinking group difference) and Y (difference in GMV, fALFF, and ReHo within the eight networks of Thomas Yeo: DMN, FPN, LIM, VAN, DAN, SMN, VSN, and SCN). (c) Differences to the module segregation index for DAN and VAN among the five groups. Binary functional connectivity matrix of each group (LA, BD, LD, LDD, and HD) versus the module segregation index (MSI) of the eight networks of Thomas Yeo in DAN and VAN. (d) Differences among the five groups for inter‐/intranetwork connectivity using the binary functional connectivity matrix. Comparison of the number of connections for inter‐/intranetworks among groups (LA vs. BD; LA vs. HD; BD vs. LD)

Based on eight networks of Thomas Yeo, MRI data showed that GMV differed across the five drinking groups with respect to the frontal–parietal–network (FPN), VAN, DAN, SMN, and VN (Figure 2a and b). ReHo and fALFF clearly differed across the networks, including the sensory control network (SCN), VN, DAN, and SMN, for the five drinking groups (Figure 2a and b).

### 3.6 Inter‐/intranetwork connectivity using binary FC matrix on the alcohol‐drinking patterns of the five groups

The binary FC matrix indicated significant differences in the MSI of DAN and VAN among the five groups (*p *< .05; Figure 2c). The binary FC matrix showed that the number of connections in the eight intra‐/internetworks for DAN and VAN significantly differed among the five groups samples (*p *< .05; Figure 2c). The number of connections in the inter‐/intranetworks of any two groups was significantly different (Figure 2d). FNP and SMN had fewer connections in BD compared to LA (Figure 2e). VAN had more inter‐/intraconnections in HD compared to LA, whereas DAN and VN had more connections in LA (Figure 2e). The left limbic network and SCN networks had more connections in BD compared to LD (Figure 2e).

### 3.7 Correlation of GMV, fALFF, and ReHo with cognition in the five groups

**FIGURE 3:**
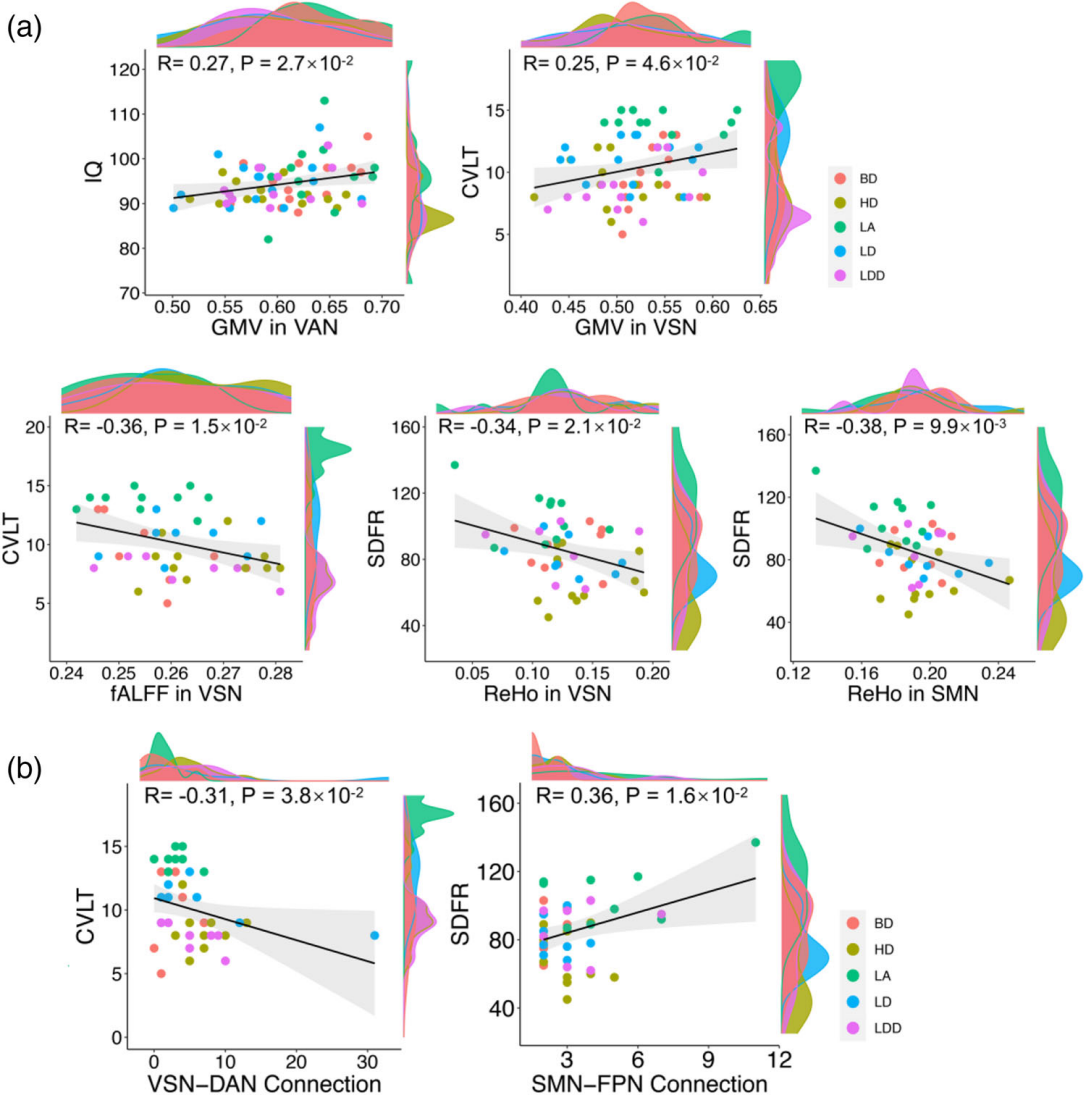
Correlation in differences to the cognitive performance of the brain and IQ in the five alcohol‐drinking groups. (a) Differences in GMV, fALFF, and ReHo correlated to brain networks with respect to cognitive performance and IQ. (b) Differences in the intraconnections of brain networks correlated with cognitive performance

The number of intra‐ and intermodular connections within networks (including VN, SMN, and VAN) and their connections to other modules differed among the five groups. These differences adversely affected cognition ability as demonstrated by various tests (e.g., IQ, CVLT, short‐delay free recall [SDFR]). Comparative analysis of BD with LD revealed that GMV and fALFF in the VN network was correlated to CVLT (learning trials correct response) scores (GMV: *r* = .25, *p* = 4.6 × 10^−2^; fALFF: *r* = −.36, *p* = 1.5 × 10^−2^). ReHo in the VN and SMN networks was correlated to SDFR scores (VN: *r* = −.34, *p* = 2.1 × 10^−2^; SMN: *r* = −.38, *p* = 9.9 × 10^−3^). GMV in the VAN network was correlated to IQ (*r* = −.27, *p* = 2.7 × 10^−2^; Figure 3a). The number of intranetwork connections between VN and DAN was correlated to CVLT (*r* = −.31, *p* = 3.8 × 10^−2^), while those between SMN and FPN were correlated to SDFR (*r* = .36, *p* = 1.6 × 10^−2^; Figure 3b).

## 4 DISCUSSION

Excessive alcohol intake is associated with structural and functional abnormalities of the brain; however, the effects of different alcohol‐drinking patterns on physical brain damage and psychosocial impairments, especially in young people, remain poorly understood. This pilot study provided new insights on this topic. The key novel findings are summarized as follows: (1) This study first showed that alcohol‐drinking patterns were significantly associated with different structural and functional disruptions of the brain in young adult drinkers. GMV mainly decreased in VAN, FPN, DAN, SMN, and VN. (2) ReHo mainly increased also mainly in VN, DAN, SMN, VN, and SCN. (3) Aberrant fALFF was detected in VN and SMN. (4) The number of intra‐ and intermodular connections of the VN, SMN, and VAN networks differed in association with different drinking patterns. Our findings on differences in the brain networks supported existing studies, and were associated with impairments in visual processing, sensor and motor adaption processing, attention processing, and memory processing. Alcohol use and misuse cause impairments to the memory, vision, and sensor and motor equipment. The current study advanced our understanding on the neural basis of ARBD.

This study clearly demonstrated that all five groups of alcohol consumption damaged the structure and functioning of the brain. These findings support previous studies showing that chronic alcohol use and misuse damages the structure and functioning of the brain. These studies proposed that to protect their brains, they must stop alcohol intake (https://www.cdc.gov/ncbddd/childdevelopment/early‐brain‐development.html). The current study provided evidence for WHO's new slogan “the less alcohol, the better” (https://www.who.int/topics/alcohol_drinking/zh/).

In particular, our data demonstrated that long‐term alcohol consumption caused structural and functional damage to the brain associated with cognitive impairments of drinkers. GMV in VN was positively correlated with cognitive performance, while GMV in the VAN was positively correlated with IQ; thus, that alcohol intake damages both IQ and cognition. However, ReHo and fALFF were enhanced in VN, SMN, and VAN, which were conversely correlated with cognitive performance. The number of connections between VN and DAN, and SMN and FPN, was negatively correlated to cognitive performance. These structural and functional differences and their relationship with cognitive performance provide scientific evidence that chronic alcohol intake causes multiple cognitive impairments.

However, in our pilot study, we demonstrated that structural damage to the brain caused by long‐term alcohol consumption impacted almost the entire brain when compared to LA samples, especially in LD, LDD, and HD groups. Of importance, long‐term alcohol consumption caused widespread structural and functional damage to the brain, regardless of the amount of alcohol consumed; thus, long‐term alcohol consumption might trigger brain damage not influenced by the total amount of alcohol intake. This suggestion needs further study for clarification. However, previous studies reporting damage to brain development in individuals exposed to alcohol prenatally supports our postulation from another perspective (Abbott et al., 2018; Feltham et al., 2020; Granato & Dering, 2018)

The GMV in the BD groups showed no significant differences to the LA and LDD groups in our study, but did differ to the LD and HD groups. The LD group had more serious GMV impairment in VAN compared to the BD group, while the HD group had more serious GMV impairment in visual network (VSN) (Figure 1a). BD is a pattern of excessive alcohol intake over a short period of time (within a 2‐h period on one occasion), with no intention to stop drinking (two to three times per month with ≥4 and 5 drinks for females and males, respectively). Although binge drinkers may consume more drinks at a time compared to LD, their alcohol‐drinking times could be lower compared to those drinkers with LD and HD patterns. In the present pilot study, the BD episodes was only twice per month and the total drinking times were low We proposed that more episodes of BD could cause more serious brain damage and this hypothesis remains to be tested in the future. In contrast to differences in structural damage to the brain among BD and LD, HD groups, significant differences in functional damage were observed in HD and BD groups compared to the LA group. The affected regions were mainly located in the VAN, SMN, VSN, and SCN. Unexpectedly, we did not observe any significant differences to functional damage among BD, LD, LDD, and HD; thus, BD and HD are more likely to induce functional impairment. We found no significant differences in GMV, functional amplitude with low‐frequency fluctuations (fALFF), or ReHo between the LDD and HD groups (Figure 1). This counterintuitive finding could not be fully explained by our study, but might be caused by the small sample size in each group. Based on an extensive literature search, we found that few studies have reported that brain damage caused by alcohol is correlated to amount of alcohol consumed, rather it is more likely to be related to personal physical fitness (Horton et al., 2015; Obad et al., 2018; Tapia‐Rojas et al., 2017), which might explain our observed differences for the LDD and HD groups. Collectively, differences in structural and functional damage to the brain among these groups were highly complex when compared each other; however, it was not possible to use the results of the pilot study to characterize the relationship between the type of damage and different drinking patterns. Despite this, our findings clearly indicated that VAN and VSN regions are structurally and functionally damaged by consuming alcohol.

The findings of the current study provide strong scientific evidence supporting the NIAAA and WHO proposal that “the less alcohol, the better” and “to protect their brains, we must stop alcohol intake.” In our study, the LA group had abstained from alcohol over a long period. All enrolled participants were grouped into five alcohol‐drinking patterns. The cumulative quantity of alcohol intake was calculated as an index to explore the relationship between alcohol‐drinking patterns and structural and functional damage to the brain. Consequently, our findings provided a detailed insight on the risk of chronic alcohol use in young adults. We showed that GMV, fALFF, ReHo, and the brain network were aberrant in participants who consumed any level of alcohol. Greater alcohol intake was positively correlated with more severe GMV fALFF, ReHo, and brain network damage. GMV, fALFF, ReHo, and brain network damage were correlated with cognitive performance. Greater alcohol intake was positively correlated with more severe cognitive impairment, especially memory function. Our findings, together with those of previous studies, demonstrate that alcohol use and misuse in young adults cause cognitive impairment, particularly with respect to memory and attention functions.

## 5 LIMITATIONS

This pilot study had several potential limitations. First, the sample size was relatively small and the major findings require validation with a larger cohort study. In the future study with a larger sample size, we need to make recruitment efforts. Specially, we consider recruiting more samples in the subgroup of LA from additional Traffic Control Bureaus where individuals are restrictedly administrated and maintain complete LA. As one of our recruitment efforts, we plan to invite multiple participating institutes to conduct the future study with larger sample size. LA samples were recruited from an administrative institute where normal social activity was restricted to all participants for more than 4 years. As such, their cognitive ability might have been influenced. Thus, more suitable LA samples should be used in future studies. Participants who were allergic to alcohol were excluded from the current study. Yet, people with allergies to alcohol are very rare, as reported by NIAAA (https://www.medicalnewstoday.com/articles/324333). Existing studies showed that participants who are allergic to alcohol exhibit unstable mood state (https://www.verywellhealth.com/do‐allergies‐affect‐your‐mood‐or‐energy‐level‐82837). Thus, inclusion of this group in future studies could enhance our understanding of alcohol on brain damage. However, small samples study should consider the effect size among different groups, which was calculated using partial eta‐squared method among different groups. As a result, the effect sizes were various from 0.89 to 0.21, suggesting statistical significance (Table 2). Second, we adopted the CVLT to assess cognitive function, as reported previously (Bell et al., 2016; Golub et al., 2015; Lee et al., 2015; Verplaetse et al., 2016). Although the CVLT has been widely used tool to assess the cognitive functions of alcohol drinkers, CVLT does not evaluate episode memory and executive function. Based on our existing data, it was not possible to quantify the differential effects of alcohol‐drinking patterns on these two factors. However, we did observe differences in SDFR scores among the five groups. SDFR can reflect impaired memory function, and that memory impairment usually causes executive function impairment (Kofler et al., 2020; Orbach et al., 2020), we postulated that the executive function of the young adult drinkers in this study might have been impaired.

**TABLE 2:** Effect sizes for GMV, fALL, ReHo, intra‐ and intermodular connections between different groups

	LA(*n* = 10)	BD(*n* = 9)	LD(*n* = 8)	LDD(*n* = 7)	HD(*n* = 11)	Partial eta‐squared
The effect size for LA versus BD (GMV)	2.52 ± 1.23	−(4.25 ± 0.98)	−(3.02 ± 1.58)	−(4.25 ± 1.28)	−(5.69 ± 1.58)	.58
The effect size for LA versus BD (fALL)	2.17 ± 1.34	−(3.89 ± 1.45)	−(4.55 ± 1.43)	−(3.82 ± 2.10)	−(4.09 ± 0.98)	.36
The effect size for LA versus BD (ReHo)	1.98 ± 0.25	−(4.14 ± 0.59)	−(3.44 ± 1.54)	−(4.74 ± 1.88)	−(5.50 ± 1.11)	.80
The effect size for LA versus BD (intra‐ and intermodular connections)	2.60 ± 1.15	−(4.89 ± 1.76)	−(2.75 ± 1.83)	−(5.71 ± 1.89)	−(5.18 ± 1.89)	.45
The effect size for LA versus LD (GMV)	2.56 ± 1.35	−(2.56 ± 1.46)	−(3.38 ± 1.17)	−(3.86 ± 1.07)	−(4.13 ± 2.64)	.21
The effect size for LA versus LD (fALL)	2.57 ± 0.97	−(3.57 ± 1.26)	−(4.75 ± 0.62)	−(4.36 ± 1.53)	−(6.13 ± 2.85)	.81
The effect size for LA versus LD (ReHo)	3.00 ± 1.11	−(5.54 ± 2.13)	−(3.75 ± 1.29)	−(3.12 ± 0.97)	−(5.70 ± 2.07)	.56
The effect size for LA versus LD (intra‐ and intermodular connections)	2.47 ± 1.57	−(5.67 ± 2.29)	−(5.88 ± 1.49)	−(3.43 ± 1.37)	−(4.40 ± 1.25)	.43
The effect size for LA versus LDD (GMV)	2.75 ± 0.45	−(3.89 ± 1.41)	−(3.08 ± 1.15)	−(4.086 ± 2.07)	−(5.13 ± 2.61)	.39
The effect size for LA versus LDD (fALL)	2.09 ± 0.78	−(2.56 ± 1.35)	−(3.25 ± 1.66)	−(4.36 ± 1.53)	−(4.78 ± 2.74)	.36
The effect size for LA versus LDD (ReHo)	2.85 ± 0.99	−(3.54 ± 0.07)	−(3.12 ± 1.92)	−(3.77 ± 1.28)	−(4.49 ± 2.33)	.25
The effect size for LA versus LDD (intra‐ and intermodular connections)	2.80 ± 2.18	−(4.67 ± 2.29)	−(3.99 ± 0.97)	−(4.77 ± 2.36)	−(5.77 ± 2.13)	.26
The effect size for LA versus BD (GMV)	2.98 ± 0.85	−(3.33 ± 1.33)	−(3.96 ± 1.88)	−(4. 60 ± 1.55)	−(6.13 ± 2.64)	.89
The effect size for LA versus BD (fALL)	1.57 ± 0.99	−(2.58 ± 1.23)	−(3.57 ± 1.11)	−(3.40 ± 1.33)	−(4.29 ± 1.61)	.33
The effect size for LA versus BD (ReHo)	2.97 ± 1.59	−(4.42 ± 0.72)	−(3.47 ± 0.99)	−(3.71 ± 1.24)	−(4.57 ± 1.15)	.27
The effect size for LA versus BD (intra‐ and intermodular connections)	2.29 ± 1.63	−(3.76 ± 1.20)	−(4.87 ± 2.09)	−(3.88 ± 1.63)	−(5.12 ± 2.22)	.35

Abbreviations: BD, binge drinking; HD, long‐term heavy drinking; LA, long‐term abstinence of alcohol; LD, long‐term alcohol drinking over safety drinking dosage; LDD, long‐term low dosage alcohol drinking.

## 6 CONCLUSIONS

This study has demonstrated the differential effects of the alcohol‐drinking patterns on the structure and function of the brain and the cognitive performance in young adult drinkers. Although limitations existed in this study, we provided new evidence supporting the need for young people to abstain from alcohol to protect their brains. In addition, the main findings may present broader implications for updating anti‐alcohol slogans for young people worldwide.

## AUTHOR CONTRIBUTIONS

Chuanjun Zhuo, Hongjun Tian, and Guangdong Chen conceptualized and designed the study. Xiaobing Guo, Tongjun Yan, and Min Chen collected the data. Xiaobing Guo, Xiaoyan Ma, Ranli Li, Bo Li, Anqu Yang, Yuhui Chen, Tao Fang, and Haiping Yu analyzed the data. Chuanjun Zhuo and Xiaobing Guo drafted the manuscript. All authors critically reviewed and finally approved the manuscript.

## CONFLICT OF INTEREST

The authors declare no financial or other conflict of interest.

### PEER REVIEW

The peer review history for this article is available at https://publons.com/publon/10.1002/brb3.2427

## Data Availability

The data that support the findings of this study are available from the corresponding author upon reasonable request.
